# Aerosol Dry Printing for SERS and Photoluminescence-Active Gold Nanostructures Preparation for Detection of Traces in Dye Mixtures

**DOI:** 10.3390/nano12030448

**Published:** 2022-01-28

**Authors:** Victor Ivanov, Anna Lizunova, Oxana Rodionova, Andrei Kostrov, Denis Kornyushin, Arseniy Aybush, Arina Golodyayeva, Alexey Efimov, Victor Nadtochenko

**Affiliations:** 1 Moscow Institute of Physics and Technology, National Research University, 141701 Dolgoprudny, Russia; ivanov.vv@mipt.ru (V.I.); kornush94@rambler.ru (D.K.); aiboosh@gmail.com (A.A.); a.golodyayeva@phystech.edu (A.G.); efimov.aa@mipt.ru (A.E.); nadtochenko@gmail.com (V.N.); 2 N.N. Semenov Federal Research Center for Chemical Physics, Russian Academy of Sciences, 119991 Moscow, Russia; oxana.rodionova@gmail.com (O.R.); andreikostrov@rambler.ru (A.K.)

**Keywords:** aerosol dry printing, gold nanoparticles, spark discharge, SERS, photoluminescence enhancement, projection on latent structures

## Abstract

We proposed a novel method of nanostructure preparation for observation of surface-enhanced Raman spectroscopy (SERS) and metal-enhanced fluorescence (MEF) based on the deposition of gold nanoparticles (GNPs) above the thin dye film by dry aerosol printing. We detected various enhanced SERS and MEF signals of films of malachite green (MG) and rhodamine B (RhB) mixtures, depending on the surface packing density of Au NPs on the strip, and found the optimum one to achieve the 3.5 × 10^5^ SERS enhancement. It was shown that statistical methods of chemometrics such as projection on latent structures provided the opportunity to distinguish SERS of MG from 100 ppm RhB in a mixture, whereas separation of MEF signals are feasible even for a mixture of MG and 1 ppm RhB due to two-photon excitation.

## 1. Introduction

Sensitive detection of trace analytes in complex environmental compounds is a pivotal demand in various fields of life ranging from food and chemical safety [1] and biological diagnostics [2] to forensic [3] and cultural heritage science [4]. For these purposes, different analytical methods are used, such as high-performance liquid chromatography (HPLC) [5], mass spectrometry [6], atomic-emission analysis [7], electrochemical stripping analysis [8], fluorescence, and FTIR spectroscopy [9,10]. In this case, the liquid phase of the analyte is requested, and on frequent occasions, the concentration of unidentified agents is raised synthetically for improvement of the detection limit or elimination of the base component influence.

In such an approach the initial samples are irretrievably destroyed, which is prohibitive in specific applications, for example, in the course of investigations of pigments and dyes in valuable paintings for the purposes of authentication and restoration.

Nowadays, innovative methods for the non-destructive characterization of works of art are proposed worldwide. For instance, a mobile laser emission spectrometer for quantitative and qualitative elemental analysis of paintings directly in the halls of museums [11] and portable digital cameras that register the ultraviolet, visible, and infrared spectral regions through external band-pass filters combined with X-ray fluorescence and fiber optic reflectance spectrometers were created for the non-invasive characterization of paintings on-site [12]. Ultra-sensitive surface-enhanced Raman spectroscopy (SERS) methodology is being developed to identify pigments in real art objects [13].

SERS is a powerful technique for the identification of traces of molecules down to the single-molecule-level [14] that can be achieved by the aid of highly sensitive SERS substrates [15], which are created by the deposition of plasmon metal nanoparticles in specific configurations on the surface of the substrate. Predominantly silver and gold nanoparticles of various sizes and shapes [16,17] and complex hybrid metal-polymer nanosized particles [18] with maximum absorption in visible spectra have been successfully used as SERS substrates to distinguish organic dyes and explosive agents in different mixtures containing up to 10 nM of the analyte [19]. Traces of malachite green in fish fillets up to concentrations of 10^−9^ M on Au nanorods coated with PNIPAM and Rhodamine B of 10^−4^ M on Ag and ZnO nanoparticle substrates have been detected by the SERS technique [20,21,22]. The creation of «hot spots», where the multiple enhancement of the electromagnetic field occurs, results in a sufficient decrease in the detection limit of the analyte compound [23].

The phenomenon of plasmon enhancement of a signal is used both in the SERS method and in metal-enhanced fluorescence (MEF) applications [24,25]. Several research groups have shown an increase of up to 10 times in the luminescence of zinc oxide near aluminum nanoparticles [26], which is characterized by localized surface plasmon resonance in the ultraviolet range [27,28]. It was shown that gold nanorods, bowties, and substrates with suspended WSe2 flakes gave the enhanced fluorescence of a weak emitter up to 1400-fold, while silicon dimers and dielectric nanoparticles have also been used as antennas in which a 270-fold fluorescence enhancement was achieved [29].

For the identification of dyes and pigments in real applications such as forensic and heritage science, where it is important to save the exterior appearance of the objects, non-destructive, highly sensitive methods of sample preparation are preferred [30]. The existing methodology and procedures for detection of low concentrations of trace analytes by SERS and MEF suggest the use of preprepared nanostructures with a dose of extracted suspensions of the real mixtures of dyes and pigments deposited above the nanostructure [31,32,33]. This means, for example, that some segment of the picture must be separated and irrevocably destroyed to complete the identification and scientific investigations. Such an approach is extremely inconvenient and impossible when it refers to valuable pieces of art and wall paintings. Therefore, in this work, we propose to consider the reverse order of sample formation for the research, where a plasmonic nanostructure is deposited on the surface of a painted layer or analyte of known composition.

Traditional methods of producing nanostructures, such as vacuum deposition and photolithography, which are multistage and include high temperature treatment and the use of aggressive resists, etchants and developers, are incompatible with the assigned task.

This is why dry aerosol printing [34], which avoids the processes of interaction of liquids with paint layers, is suggested to be a promising technology for developing nanostructures on the surface of paintings and dyes. Aerosol metal nanoparticles produced in a gas discharge generator [35,36] can be used as a source for the formation of nanostructures. The implication of additional in-flow thermal [37] or the laser treatment [38] of aerosol nanoparticles leads to varied morphology, as well as optical properties, of nanoparticles which is extremely important for the fabrication of sensitive SERS and luminescence substrates to be applied. Beyond that, after SERS and MEF investigations, the nanostructures based on bare aerosol nanoparticles without any surfactants on their surfaces can be removed from a sample by a pure gas jet (without nanoparticles) passed through the nozzle for aerosol printing without damaging the painted layer.

Thus, the goal of this research is to fabricate gold nanoparticles strips (GNPS) using the original, non-traditional method of sample preparation for SERS, which is based on dry aerosol printing gold nanoparticles onto the surface of dye/painted films. Then, to investigate the ability of the obtained strips to enhance SERS and luminescence signals for detection of small amount of analyte in the mixture, mixtures of xanthene dye Rhodamine B (RhB), as a small impurity in the film of the triarylmethane dye malachite green (MG), is used as a model system. MG is an effective quencher of RhB fluorescence in liquid solutions. At the same time, MG has a low quantum yield of luminescence in fluid liquids, but it demonstrates moderate luminescence in solid films. The SERS effect of GNPS was examined for near resonance and off resonance of RhB excitation. The effect of GNPS on the luminescence of the dye mixture of RhB and MG was tested under the conditions of two-photon excitation with femtosecond pulses of 976 nm and single-photon excitation at 488 or 561 nm.

## 2. Materials and Methods

Solutions of malachite green (NILPA, Moscow, Russia) and Rhodamin B (Sigma-Aldrich, New York, NY, USA) were prepared in chromatography 2-propanol (LiChrosol, Darmstadt, Germany) without further purification, with a concentration of 10 and 1 g/L, correspondingly. Then, mixtures of dyes with mass fractions of 1, 10, and 100 ppm of Rodamin B diluted in malachite green were fabricated by simple mixing. To get thin films of the dyes’ mixtures, one drop (2 µL of solution) was deposited on the surface of quartz or indium tin oxide (ITO) glasses (1 × 1 cm^2^) until the films were completely dry, in air atmosphere, for SERS (Bruker, Berlin, Germany) and scanning electron microscopy (SEM, Waltham, MA, USA) investigations, respectively. To achieve the homogeneity of the film thickness, the glass substrate was periodically slowly turned when the solution reached the edge of the substrate.

The formation of nanoparticle arrays in the form of strips on the surface of the obtained dye films was carried out using an experimental setup, as shown in [34]. It includes the following key elements: a spark discharge generator of nanoparticles, a thermal nanoparticle optimizer to control the size and shape of the nanoparticles, a coaxial micro-nozzle to deposit the nanoparticles on a substrate, and a coordinate table to move the substrate at a given speed. Nanoparticle arrays are formed in dry form without using solvents and surfactants.

In the spark discharge generator, the nanoparticles were synthesized by the electrical erosion of gold electrodes in an argon–hydrogen flow (Ar 95% + H_2_ 5%). In the thermal optimizer with a temperature of 950 °C, the synthesized gold nanoparticles changed their size and acquired a spherical-like shape in the heated atmosphere [36]. Further, the optimized gold nanoparticles were focused and deposited on the substrate through a coaxial micro-nozzle with a diameter of 100 μm. The focused deposition of nanoparticles was controlled by an aerosol flow Qa and a sheath flow Qsh. The sheath flow Qsh also prevented the nozzle from clogging. The distance from the nozzle to the substrate was 9 mm. The formation rate of nanoparticle arrays in the form of strips was varied from 0.07 to 1.6 mm/s (high–low range 7 µm/s–140 mm/s with step 7 µm/s). The number of the obtained strips and the corresponding formation rate are presented in Table 1. The length of the strips was 5 mm. It is worth noticing that the previously deposited nanoparticles could be easily blown off by the clean gas flow (without nanoparticles) running from the nozzle.

The thickness of the dry dye films was measured by Sensofar S neox optical profilometer. The microstructure of the strips, the morphology, and the size of the gold nanoparticles deposited on the surface of dyes were investigated by the scanning electron microscope Prisma E SEM from Thermo Fisher Scientific (Waltham, MA, USA). SERS measurements were performed using a Senterra Raman microscope (Bruker, Berlin, Germany) with the following parameters: (1) excitation wavelength 785 nm, which is beyond the absorption peaks of both MG and RhB, excluding the mainly complex energy interplay between pigments though the field enhancement factors of plasmonic NPs (which can still be exploited); and (2) the mode of the minimum available laser power of 1 mW with the simultaneous use of a microscopic objective with a low numerical aperture (0.25) to eliminate bleaching effects, as well as to more effectively deal with surface heterogeneity on a submicron level. Laser scanning microscope (LSM) studies were performed on the LSM-980 multicolor laser scanning microscope (Zeiss, Berlin, Germany). The measurements were made using a sensitive GaAsP PMT detector, which allowed working with laser powers of microwatt scale, as well as making multiple lambda scans of the same sample area without any bleaching. The excitation wavelengths were chosen to be: (1) 561 nm, which is close both to LSPR position and absorption maxima of RhB; (2) 488 nm, which is close to the absorption deep of MG; and (3) 976 nm (femtosecond pulses of Discovery-NX, Coherent), which is far from the absorption bands of both pigments and utilizes their ability for multiphoton luminescence with the aid of a plasmonic subsystem.

Projection on latent structures (PLS) [38] was used for statistically processing the SERS spectra. This method performs decomposition of the matrices, the X-predictors and Y-response, simultaneously in order to maximize the correlation between the corresponding vectors of X-scores and Y-scores. We considered a set of 160 spectra as the X-matrix, namely, 74 spectra were used for a mixture of 1 ppm rhodamine, 53 spectra to analyze a 10 ppm RhB mixture in MG, and 35 signals of 100 ppm RhB. 119 spectra from all mixtures with different concentrations of rhodamine were selected for model calibration and 41 spectra were collected in the test set for model validation. The pre-processing of spectra consisted of (1) standard normal variate (SNV) correction for spectra scaling; (2) averaging by two wavelengths (for reduction of the number of variables from 2301 to 1151) for convenience; and (3) column-wise centering as a common procedure for projection methods.

## 3. Results

### 3.1. Structure and Spectra of GNPS on RhB and MG Paint

The dye films were substantially uniform over the surface of the substrates, regardless the rhodamine B concentration. The average thickness measured by optical profilometer was 220 ± 35 nm.

**Figure 1:**
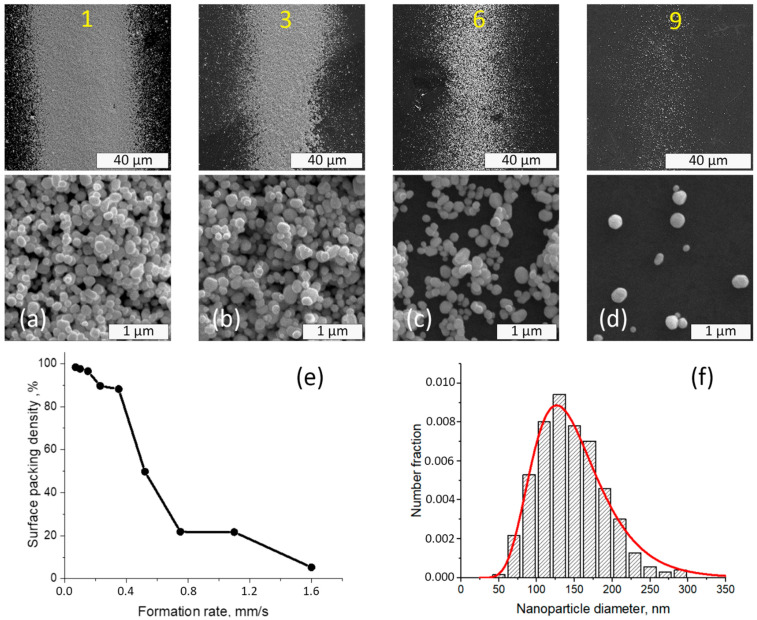
SEM analysis of the surface of the strips on dye mixture film with 10 ppm of RhB. General SEM images (upper line) and microstructure of (**a**) 1st, (**b**) 3rd, (**c**) 6th, and (**d**) 9th strips’ surface (middle line); (**e**) the dependence of surface packing density of nanoparticles on the formation rate of the nozzle; and (**f**) the particle size distribution approximated by lognormal function. Yellow numbers correspond to the numeration of GNPS.

The surface packing density of nanoparticles on the dye films was influenced by the formation conditions of the strips for all films, independent of rhodamine B concentration. The typical microstructure of strips of gold nanoparticles deposited on dye films is presented in Figure 1. The increase in the formation rate led to a decline in the area of surface that was filled by the nanoparticles and a decreased width of the line. The size distribution of gold nanoparticles produced by the spark discharge was calculated from SEM image analysis and is shown in Figure 1f. The particle size varied from 50 to 300 nm, and the average size of gold nanoparticles was 144 ± 44 nm.

**Figure 2:**
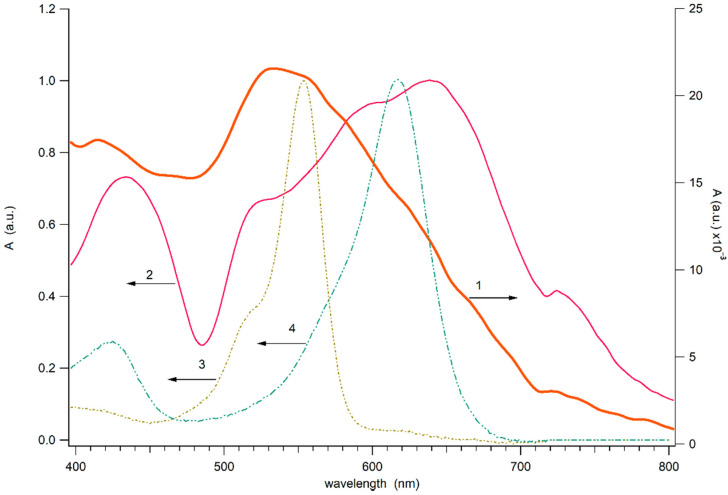
Absorbance of dense Au NPs strip on the net glass (1) and on the glass painted by the mixture of RhB and MG (2) (1 ppm). The absorbance of the glass covered by RhB (3) and MG (4) film.

Figure 2 demonstrates the absorbance (absorbance plus scattering) spectrum of the close-packed Au NPs strip (with condition of the deposition similar to strip 1) on the glass substrate and spectra of the dense Au NPs strip on deposited on the glass covered by dye layer. For comparison, Figure 2 (3,4) shows the dye layers of clean RhB and MG films on the glass. The spectrum of the strip differs significantly from the spectrum of an individual gold nanoparticle, which is characterized by pronounced plasmon resonance. The absorption spectrum of the close-packed strip of gold nanoparticles on the glass surface appears as a broad band extending into the near-IR range. The broadening of the plasmon resonance spectrum during the formation of an agglomerate of nanoparticles was previously recorded and is associated with the dipole–dipole interaction between closely spaced plasmon nanoparticles [39]. The bandwidth of the spectrum of the band on clean glass is somewhat narrower than that of the band on glass painted with dyes. This effect may be due to the fact that the dye layer has a dielectric constant that differs from that of glass, which affects the plasmon resonance of the nanoparticles. However, it cannot be ruled out that when Au NPs are applied to the surface, the mechanical properties of pure and painted glass differ, which affects the formation of agglomerates of gold nanoparticles in the strip.

This spectral feature of the strip of Au NPs is of interest for obtaining the Raman spectrum of light when excited by a 785 nm laser in the near-IR range since this wavelength falls into the spectrum of the electromagnetic field of the plasmon system. At the same time, the wavelength of 785 nm is rather far from the resonance bands of dyes and, at a moderate laser power, does not excite dye fluorescence undesirable for Raman spectroscopy. Figure 2 also shows the absorption spectra of two dyes applied with a thin layer of paint on the glass surface. The spectra of RhB and MG dry layers are somewhat different from the spectra of these dyes in solution (see Supplementary Information Figure S1). Apparently, the difference is related to close packing and intermolecular interactions between dye molecules. As can be seen from Figure 2, the dyes have no absorption at 785 nm.

### 3.2. SERS Effect of GNPS on RhB and MG Dyes

The wavelengths of the SERS excitation sources were 785 and 532 nm. On the one hand, the wavelength of 785 nm is distant from the plasmon absorption bands of dyes; on the other hand, it is contained within the infrared tail of the GNPS plasmon resonance band (Figure 2). Thus, non-resonant Raman scattering of dyes is expected under infrared excitation. On the other hand, the 532 nm source is close to the absorption peaks of both dyes and falls into the GNPS plasmon resonance band, so resonant Raman scattering can be observed.

The SERS effect greatly depends on near-field distribution around gold nanoparticles and formation of “hot spots” on the surface. Since GNPS on dye mixture films of RhB and MG have the form of disordered particle aggregates, SERS spectra were measured at 11 randomly selected points for each experimental strip. Raman spectra without SERS was measured on the 11 random points of RhB and MG film outside the GNPs strip. The enhancement was determined as the ratio of the averaged area under the Raman spectra curves with and without SERS (Figure S2).

**Figure 3:**
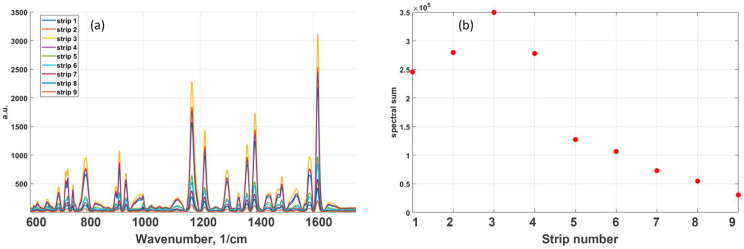
SERS signal of MG and RhB 10 ppm dye mixture obtained at 785 nm excitation wavelength: (**a**) effect of gold NPs surface packing density on the SERS spectra; and (**b**) the dependence of SERS enhancement on NPs packing density.

Figure 3a demonstrates the SERS spectra for a mixture of dyes (10 ppm RhB) under GNPs strips with different surface packing densities of gold particles on the film. Figure 3b shows the existence of an optimal density of nanoparticles which achieved the maximal SERS enhancement. As seen in Figure 3, GNPs strip spectral sum enhancement was found to be 3.5 × 10^5^, while decreasing GNPs strip density expectedly diminished SERS enhancement. For the first and second lines with higher GNPS density, the upper layers of gold nanoparticles can shield the down layer located in contact with the dye film, which can also lead to a signal decrease.

**Figure 4:**
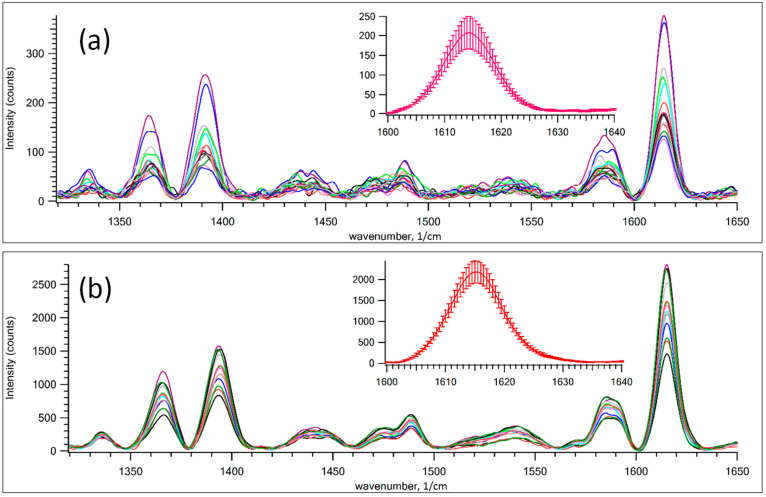
SERS of a mixture of 10 ppm RhB and MG with Raman scattering of 785 nm. The effect of hot spots distribution on SERS signal for GNPS number 1 and 9: (**a**) GNPS 9, low-density strip; and (**b**) GNPS 1, high-density strip.

Figure 4 shows the SERS signal of MG and 10 ppm of RhB dye film measured at different spots on GNPS. GNPS 1 is a high-density strip whereas GNPS 9 corresponds to a low-density strip prepared by fast aerosol printing. Inserts show the signal of the Raman band of 1615 cm^−1^. It was obtained by averaging all spectra from subplots. Error bars qualitatively suggest the distribution of hot spots in the GNPS. As expected, in a close-packed band, the relative error of the averaged spectrum is lower than in a less dense band. This observation suggests that the distribution of hot spots is wider in low-density films.

**Figure 5:**
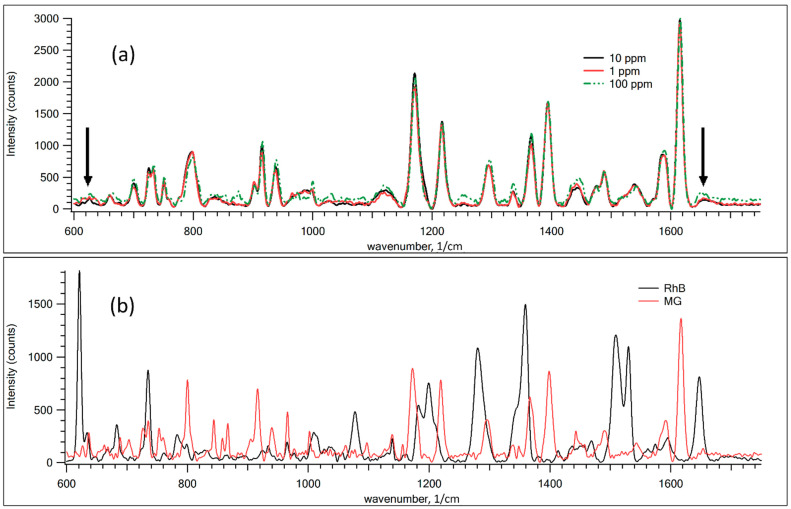
(**a**) SERS Raman spectra for mixtures of RhB and MG samples with different RhB concentrations on GNPS. (**b**) Raman spectra of pure RhB and MG films. The black arrows mark the main distinguishable peaks of RhB in the mixtures.

Figure 5 demonstrates the SERS spectra for various dye mixtures. For comparison, Figure 5b shows the Raman spectra of pure RhB and MG powder. As shown in Figure 5a, the discrepancy in the SERS spectra for different concentrations of RhB from 1 to 100 ppm is negligible in recognizing whether there is a statistically significant difference between the samples. PLS analysis was performed to show the ability to define the small traces of RhB in mixtures.

The dependence of the enhanced Raman signal on the density of the nanoparticle layer per dye layer, shown in Figure 3, and the change in signal intensity shown in Figure 4 are due to the features of the plasmon mode in the random structure of plasmonic Au NPs [40,41,42,43,44]. The enhancement of the Raman scattering near a spherical nanoparticle depends on the diameter of the nanoparticle and the distance between nanoparticles and analyte [45,46]. It is shown that the deposition of nanoparticles above the MG leads to an increase in the value of the registered SERS signal up to 14 times in comparison with the analyte deposition on top of the metal nanostructure. It is noticeable that the use of a sandwich configuration, in which the MG is deposited between layers of plasmonic nanoparticles, can provide an additional Raman enhancement factor of up to five times compared to the intensity of the SERS signal of the analyte, where the analyte is under the metal film configuration [47]. The size distribution of nanoparticles shown in Figure 1e affects the variation of the SERS signal. However, the distance of the spatial gap between the randomly distributed nanoparticles and the corresponding gap–plasmon seems to have a stronger effect on the SERS signal [48,49]. The magnitude of the gap-mode plasmon is known to be inversely proportional to the gap distance between two metal nanoparticles. This assumption is consistent with the fact that the SERS signal drops with a decrease in the density of nanoparticles on the painted surface (Figure 3b). The observed decrease in the intensity of the Raman signal, noted for the first two strips (strip 1, strip 2, Figure 3b) with the closest packed density of nanoparticles, is apparently because excessive compaction or thickening of the layer of nanoparticles leads to darkening of the lower layer contacting with the dye film. It means that the Au NP in upper layer shields nanoparticles located in the lower one.

### 3.3. Results of PLS Modeling

**Figure 6:**
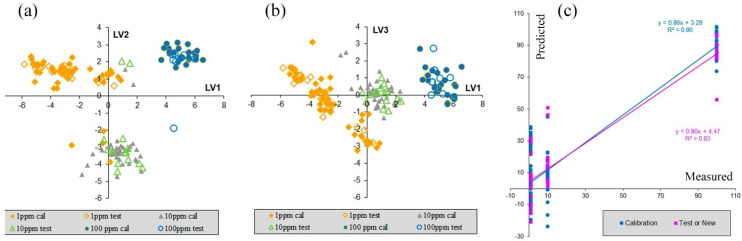
Results of PLS calculations: (**a**,**b**) PLS model with three LVs. The filled markers refer to calibration samples and the open markers refer to test samples. (**c**) PLS model with two LVs: the predicted vs. measurements plot for calibration and test samples.

The results of the PLS calibration are presented in Figure 6. Score plots for various latent variables (LVs) show the division of samples into three groups, which present spectra with three different RhB concentrations. This grouping is better seen in the LV1 vs. LV3 score plot and refers both to calibration and test samples.

The prediction vs. measurement plot (Figure 6c) shows that accuracy of y-prediction for samples of 1 ppm concentration of RhB is rather low. The corresponding values are a comparison to those of samples with 10 ppm concentration. Thus, we can conclude that samples with 100 RhB ppm concentration can be found and probably quantitatively predicted. The results of PLS calibration also show that samples with concentrations of 10 ppm, and even of 1 ppm, probably could be detected, but additional experiments with several different concentrations in the range of 1 ppm to 10 ppm are required.

### 3.4. Luminescence Studies

**Figure 7:**
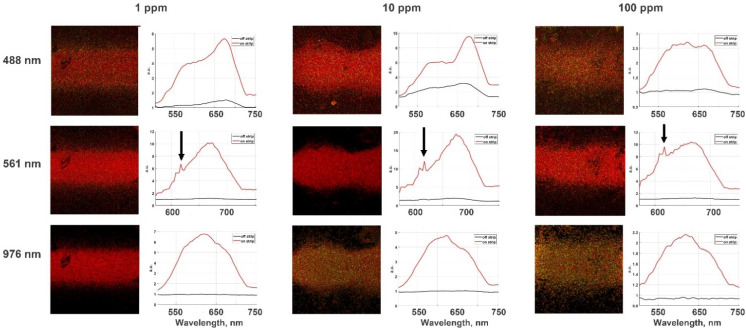
Luminescence spectra exited by 488, 561 (LED), and 976 nm (two-photon excitation by a femtosecond laser) on films with different amount of RhB (1, 10, and 100 ppm) in MG; the arrow marks the Raman bands for a Raman shift of 561 nm. The black lines indicate a luminescent signal on the dye’s mixture outside the strip, while the red lines on PL spectra show the PL signal of dye mixtures under the gold nanoparticles.

Figure 7 shows confocal microscope images of the films with various dye mixtures covered by aerosol gold NPs strips deposited above the film and under the radiation excitation of three different wavelengths: 488, 561 and 976 nm. The obtained luminescence spectra for the corresponding films are represented on the right of the images.

The results shown in Figure 7 demonstrate significant phenomena for various excitation wavelengths. Firstly, amplification of luminescence occurs in both cases: during the excitation approximately in the absorption band of dyes and also under the infrared radiation according to the two-photon mechanism (see Figure S1). The calculated photoluminescence enhancement is approximately 10 times. Secondly, the ratio of the intensity of a photoluminescence peak at the position of about 620 nm associated with rhodamine B to the intensity of the malachite green PL peak located at the position about 720 nm depends on the excitation wavelength (see Figure S1).

Since the preparation method of the dye films ensures their uniformity, the difference in the shape of the luminescence spectra cannot be associated with the inhomogeneous distribution of rhodamine B molecules in the dye films on the substrate. The shape of the PL spectra is influenced by the distinction between the enhancement factor for malachite green and rhodamine B at different excitation wavelengths. MG is known to be an effective RhB quencher. Nevertheless, RhB luminescence is observed. In a mixture of RhB and MG, the near-field electromagnetic behavior around the plasmonic structure in various regions encourages the luminescence of the two dyes in different ways. Malachite green (MG) is an organic dye with almost no fluorescence when in solution by itself with a quantum yield of 7.9 × 10^−5^. Stark [50] and Schmidt [51] have noticed that some diphenyl- and triphenyl-methane dyes which do not fluoresce in ordinary solvents will, however, fluoresce strongly in highly viscous media, such as glycerol, at low temperatures. Thus, photoluminescence of MG in dry film can be detected. MG is an effective quencher of RhB luminescence, as was already reported [52,53,54,55].

**Figure 8:**
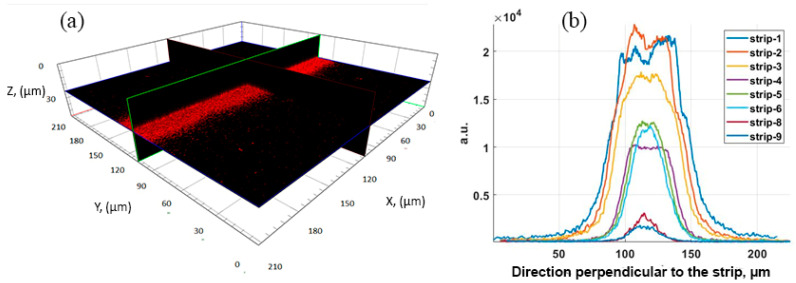
Enhancement luminescence effect of GNP strips: (**a**) 3D image of strip 1 (left) and (**b**) integral MEF profile for 580–780 nm on the different strips deposited on the dye mixture with 10 ppm of RhB. Excitation at 561 nm.

Figure 8 shows a 3D confocal microscope image of a strip of Au NPs on the dye surface and the luminescence signal for strips with different surface densities of deposited Au NPs. The luminescence signal on the strips with a low packing density of Au NPs is expected to decrease. For strip 1, with the highest close-packing density of Au NPs, the signal does not exceed, and is even somewhat lower, than for strip 2, with a slightly lower Au NPs density. To assess the amplification effect, the ratio of the background signal (in black) to the luminescence signal (in red) was used. So, the calculated enhancement factor is influenced by the rhodamine concentration and ranges from ~7 to ~20 for the strips with the maximum signal amplification.

## 4. Discussion

Simultaneous observation of the Raman and fluorescence enhancement of dyes under the strip of randomly closed packed gold nanoparticles means that an intense electromagnetic field through the strip was generated. The interaction of a metal nanostructure with electromagnetic radiation is markedly determined by the free conduction electrons in a metal, which are dedicated by localized surface plasmons [40,56]. Surface plasmons are collective electromagnetic modes which strongly depend on the morphology of the metal surface. By the action of optical excitation, the behavior of nanostructures with randomly arranged nanoparticles significantly differs from nanostructures with a regular grid, such as metal nanolattices, nanoscale matrices, and substrates from arrays of nanorods with compact or periodic geometry [57,58,59,60,61]. The excitation of surface plasmon propagated at the metal–dielectric or metal–air interfaces occurs in the form of plane traveling waves, which are usually delocalized over large areas. On the contrary, metallic nanostructures of randomly and close packed nanoparticles do not possess translational invariance and, therefore, cannot form traveling waves. This fact results in the localization of plasmon modes in nanoparticle assemblages, which are localized in small nanometer-sized regions and form hot spots [39]. The local enhancement of an electromagnetic field in “hot spots” can be many orders of magnitude higher than the average surface amplification in the vicinity of an individual nanoparticle due to large, transient surface dipoles induced by plasmon resonance.

An explanation of the effect of SERS and MEF under a strip of randomly and closed packed Au nanoparticles is based on the mechanism of the electromagnetic field (EM) enhancement in hot spots [62,63,64]. To be precise, the factor of PL enhancement is proportional to the quantum yield modified by the proximity of a metal nanostructure and the intensity of a localized electromagnetic field in the nanostructure. Thus, the enhancement factor for fluorescence of low quantum yield emitters can obey the same form as for SERS [46]. For an individual nanoparticle, the SERS and MEF cross section σ_SERS,MEF_ (λ_ex_, λ_em_, d) can be expressed analytically as a function of the excitation wavelength λex, the radiation wavelength λ_ex_, and the distance d between the molecule and the surface of a metal nanoparticle. The significant SERS and MEF cross section σ_SERS,MEF_ (λ_ex_, λ_em_, d) is achieved by the amplification of the EM field in “hot spots”, which is formalized through the enhancement factor of the EM field MEM (λ_ex_, λ_em_, d) [64,65,66].

Since, in our experiments, we have randomly packed nanoparticles on the surface, the parameter d is difficult to formalize and we are restricted to the consideration of only the expression of MEM (λ_ex_, λ_em_).

Let |MEM|^2^ be the overall EM enhancement factor:

|MEM(λ_ex,_ λ_em_)|^2^ = |M1(λ_ex_)|^2^ × |M2(λ_em_)|^2^ (1)

where factors |M1(λ_ex_)|^2^ = |E_hot spot_ (λ_ex_)/E_in_ (λ_ex_)|^2^ and |M2|^2^ = |E_hot spot_ (λ_em_)/E_in_ (λ_em_)|^2^ are EM enhancement factors corresponding to the interaction of the plasmon resonance with incident light λ_ex_, (|M1(λ_ex_)|^2^) and Raman scattering of light, or fluorescent light λ_em_, (|M2(λ_em_)|^2^), respectively.

In the case of MEF σ_MEF_ (λ_ex,_ λ_em_), the fluorescence decay rate can also increase by a factor of |Md(λ_em_, d)|^2^ times due to the transfer of energy from the molecule to the metal surface [54,55,56]. The cross section can be expressed as σ_MEF_ (λ_ex,_ λ_em_) = σ_FL_ (λ_ex,_ λ_em_) × |MEM(λ_ex,_ λ_em_)|^2^/|Md(λ_em_, d)|^2^.

Thus, according to the EM model of amplification, the SERS cross section will be expressed as σ_SERS_ (λ_ex_, λ_RS_)~|MEM|^2^ σ_RS_ (λ_ex_, λ_RS_).

The experiment showed that both the SERS effect and the MEF are enhanced under a strip of randomly closed packed Au nanoparticles. Moreover, the SEF effect amplificated itself both in the case of single-photon excitation at wavelengths of 488 and 561 nm and with two-photon excitation at 976 nm. Since the overall enhancement factors |MEM|^2^ and the coefficient |Md(λ_em_, d)|^2^ depend on the wavelengths λ_ex_ and λ_em_, this circumstance can be regarded as a qualitative explanation of the dependence of the envelope curve for luminescence spectrum on the excitation wavelength.

## 5. Conclusions

The novel method of nanostructure preparation was developed to get the SERS and enhanced photoluminescence signals. The proposed technique is predicated on the aerosol printing deposition of 144 nm gold nanoparticle strips above the thick dye films prepared from a solution of mixtures of 1, 10, and 100 ppm rhodamine B in malachite green. This method can have a significant advantage for the identification of dyes and pigments in real applications, such as heritage arts, due to the deposition of nanoparticles above the paint and usage of the highly sensitive PL and SERS techniques directly on the art object without sample selection. That fact leads to the detection of traces of dyes with further non-destructive removal of nanoparticles from the object of art, resulting in its conservation for the next generation in its initial conditions.

It was found that the SERS effect depends on the location of the hot spots and the surface packing density of metal nanoparticles on the dye film. The enhancement factor of 3.5 × 10^5^ was achieved on the strip with a surface packing density of 97%. Only 100 ppm rhodamine mixed with malachite can be proven to be distinguished in SERS spectra by statistical processing using the projection on latent structures procedure, whereas, in the luminescence spectra, it is possible to detect the enhancement effect and separate malachite and rhodamine even at a concentration of 1 ppm by the aid of multi-photon excitation.

## Figures and Tables

**Table 1:** The formation conditions of the strips.

Number of a Strip	1	2	3	4	5	6	7	8	9
Formation rate mm/s	0.07	0.1	0.15	0.23	0.35	0.52	0.75	1.1	1.6

## Data Availability

Not applicable.

## Supplementary Materials

The following supporting information can be downloaded at: https://www.mdpi.com/article/10.3390/nano12030448/s1, Figure S1: Normalized absorbance and fluorescence spectra; Figure S2: SERS signal of MG and RhB 10 ppm dye film under excitation at 785 nm measured from the GNP strip 1 (red curve) and on the dye film surface without gold nanostructure (black curve).
