# Supplementary material for: Aerosol Dry Printing for SERS and Photoluminescence-Active Gold Nanostructures Preparation for Detection of Traces in Dye Mixtures
Source: Nanomaterials (Basel). 2022 Jan 28;12(3):448. doi: 10.3390/nano12030448 (PMC8840613; doi:10.3390/nano12030448)
Supplement: Supplementary file 1 [file nanomaterials-12-00448-s001.zip › nanomaterials-1569892-supplementary.pdf]

Supplementary Materials

Aerosol Dry Printing for SERS and Photoluminescence-Active Gold Nanostructures Preparation for Detection of Traces in Dye Mixtures

Victor Ivanov [1], Anna Lizunova [1,*], Oxana Rodionova [2], Andrei Kostrov [2], Denis Kornyushin [1], Arseniy Aybush [1,2], Arina Golodyayeva [1], Alexey Efimov [1] and Victor Nadtochenko [1,2]

[1] Moscow Institute of Physics and Technology, National Research University, 141701 Dolgoprudny, Russia; ivanov.vv@mipt.ru (V.I.); kornush94@rambler.ru (D.K.); aiboosh@gmail.com (A.A.); a.golodyayeva@phystech.edu (A.G.); efimov.aa@mipt.ru (A.E.); nadtochenko@gmail.com (V.N.)

[2] N.N. Semenov Federal Research Center for Chemical Physics, Russian Academy of Sciences, 119991 Moscow, Russia; oxana.rodionova@gmail.com (O.R.); andreikostrov@rambler.ru (A.K.)

* Correspondence: lizunova.aa@mipt.ru

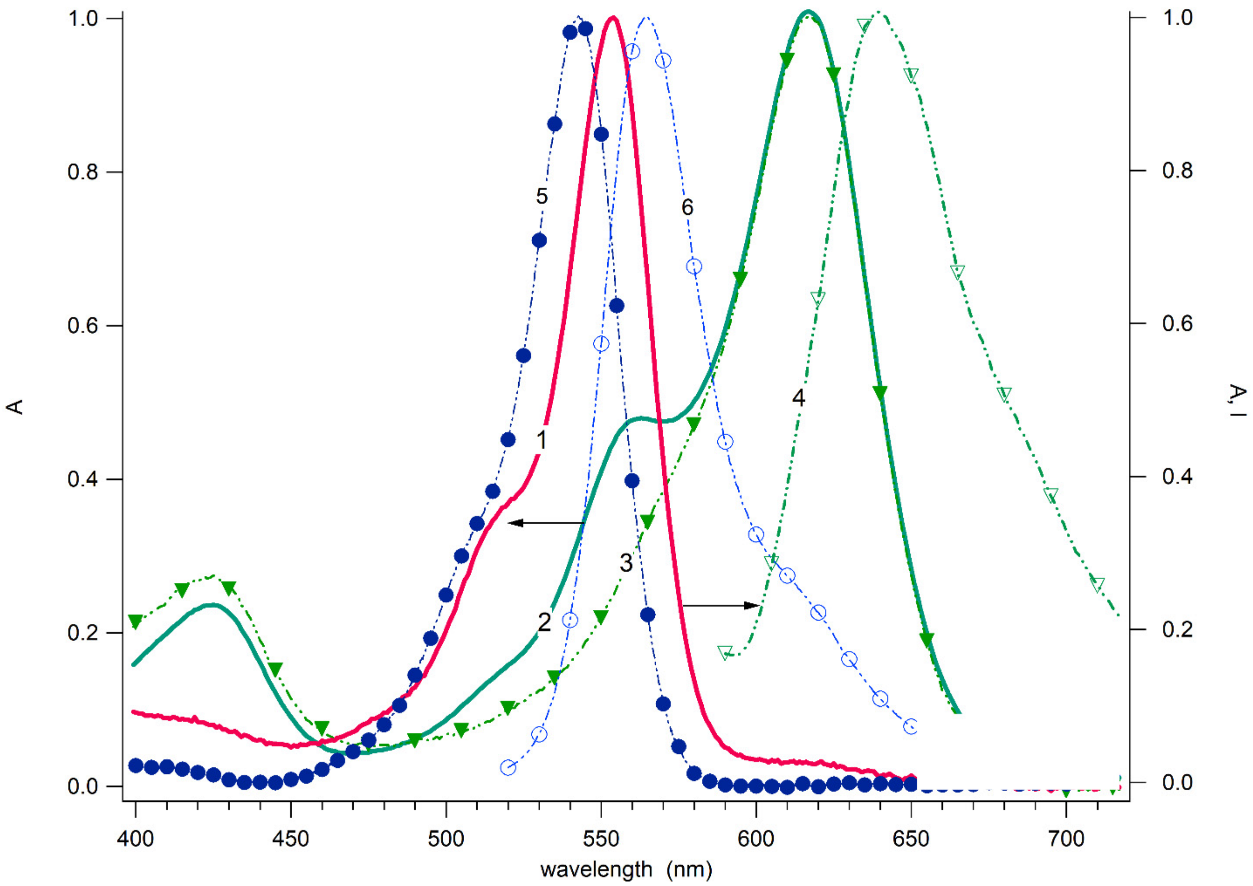

Figure S1. Normalized absorbance and fluorescence spectra:

1. Absorbance. Dry RhB layer on the glass substrate
2. Absorbance. Dry MG layer on the glass substrate
3. Absorbance. MG in ethanol solution (30 μM)
4. Fluorescence. Dry MG layer on the glass substrate
5. Absorbance. RhB in ethanol solution (45 μM)
6. Fluorescence. RhB in ethanol solution (45 μM)

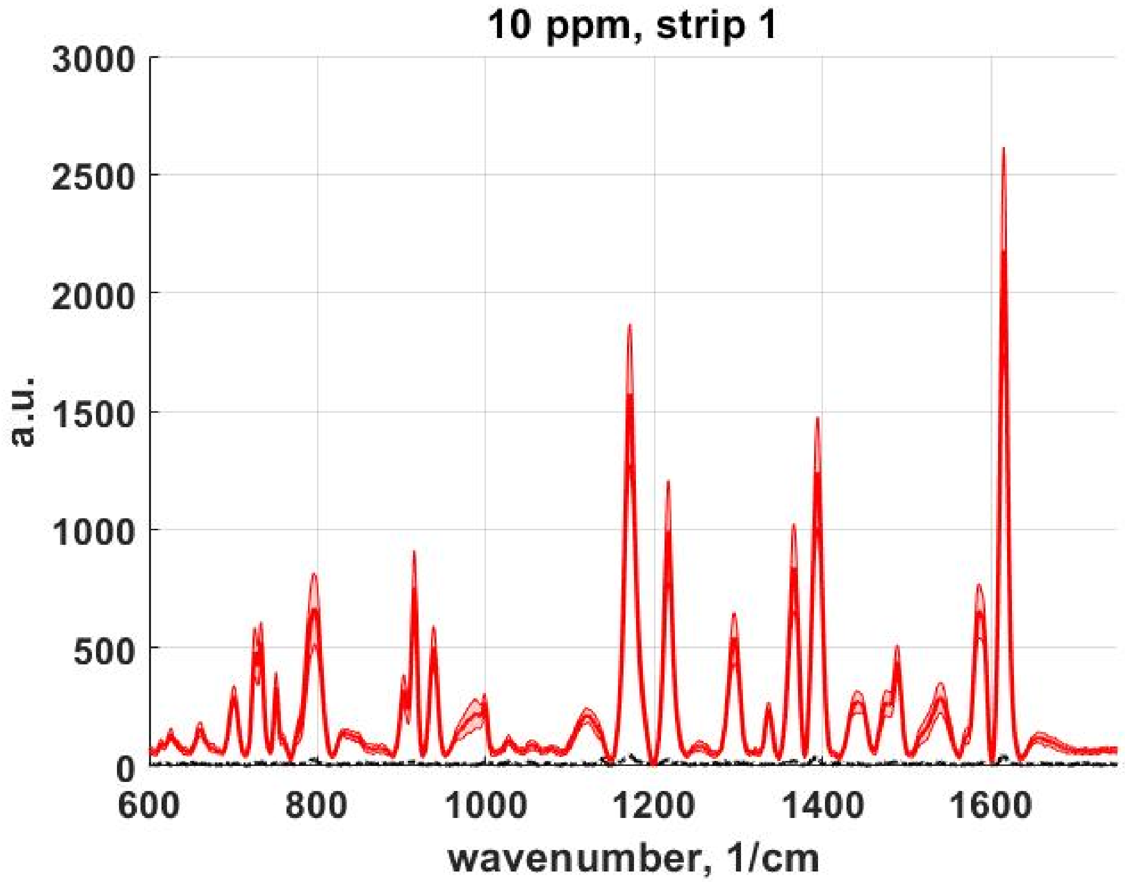

Figure S2. SERS signal of MG and RhB 10 ppm dye film under excitation at 785 nm measured from the GNP strip 1 (red curve) and on the dye film surface without gold nanostructure (black curve).
